# The intergenerational persistence of opioid dependence: Evidence from administrative data

**DOI:** 10.1002/hec.4589

**Published:** 2022-08-15

**Authors:** Alexander Ahammer, Martin Halla

**Affiliations:** ^1^ Department of Economics Johannes Kepler University Linz Austria; ^2^ Christian Doppler Laboratory Aging, Health, and the Labor Market Linz Austria; ^3^ IZA, Institute for the Study of Labor Bonn Germany; ^4^ GÖG, Austrian Public Health Institute Vienna Austria

**Keywords:** addiction, drug abuse, heroin, illicit opioids, intergenerational correlation, intergenerational transmission, opioids, prescription opioids

## Abstract

To address the opioid crisis, it is crucial to understand its origins. We provide descriptive evidence for the intergenerational persistence of opioid dependence. Our analysis is based on administrative data covering the universe of Austrian births from 1984 to 1990. We consider prescription opioids and a new proxy for addiction to illicit opioids. We find that, if at least one parent is using illicit opioids, the likelihood of the child using increases from 1% to 7%. For prescription opioids, we observe an increase from 3.6% to 6.7%. Both associations are stable and do not change when controlling for environmental variables.

## 1 INTRODUCTION

Opioid dependence, misuse, and overdoses are serious public health problems faced by many countries. Particularly in the United States (US) and Canada, the use of opioids has surged since the late 1990s. This trend is observed for both illicit opioids, such as heroin, and prescription opioids. Today, both countries are in the midst of a devastating opioid epidemic, which is likely to become even worse during the COVID‐19 pandemic.^1^ Some observers are worried that this epidemic could soon swap over to other countries.^2^ In Europe, overdose deaths have recently begun to increase again too. Especially the emergence of potent synthetic opioids, such as fentanyl, is alarming (EMCDDA, 2017). To address this crisis and understand its persistence, it is crucial to identify important predictors of opioid dependence.

Opioid dependence not only has a negative impact on users themselves but harms entire families. Children are particularly vulnerable. The incidence of neonatal abstinence syndrome, a condition that occurs if babies are exposed to opioids in utero, has increased almost fourfold between 2004 and 2013 (Tolia et al., 2015). Affected babies experience severe withdrawal symptoms up to 6 months after birth (McQueen & Murphy‐Oikonen, 2016) and are more likely to have adverse outcomes in later life (Maguire et al., 2016). Children exposed to parental opioid dependence postnatally face obstacles as well. They are more likely to grow up in an unstable environment with economic and emotional challenges, such as secrecy, loss, conflict, violence, and fear (Nunes et al., 2000). Such childhood experiences are associated with severe limitations in economic and social functioning later in life, which may increase the likelihood of children's own substance abuse. Thus, the family is potentially an important factor in explaining opioid addiction.

**FIGURE A1:**
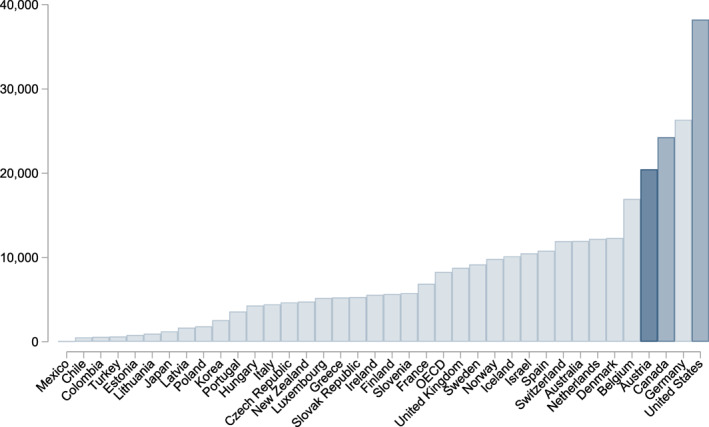
Opioid prescriptions per million population among OECD member countries. Average availability of analgesic opioids in OECD countries 2014–2016 in defined daily doses for statistical purposes per million population per day. The data are retrieved from www.oecd.org/els/health‐systems/opioids.htm, accessed on June 16, 2020

This presumption is in line with a vast literature on substance abuse, which concludes that child addiction is often linked to parental addiction. The association between parental and child substance abuse has been shown for alcohol (Kendler et al., 2015; Schmidt & Tauchmann, 2011; Walters, 2002), smoking (Göhlmann et al., 2010; Leonardi‐Bee et al., 2011; Mays et al., 2014; Melchior et al., 2010), and cannabis (Henry & Augustyn, 2017; Roettger et al., 2011). However, very little is known about the case of opioids. We fill this gap by providing credible estimates of the intergenerational persistence of opioid dependence. We use administrative data from Austria, which combines several useful features. First, while Austria has not been experiencing an opioid crisis comparable to the US, opioid use is nonetheless very high. Austria ranks fourth in opioid prescriptions per million population among OECD countries (see Figure A1). Second, we can track the vast majority of opioid users in statutory health insurance data and do not have to rely on survey measures. Importantly, 99% of Austrian residents enjoy full healthcare coverage (Ahammer et al., 2021), so we also observe people who do not participate in the labor market. Third, we are not only able to identify the users of prescription opioids, but we can also observe former and current users of illicit opioids, such as heroin. As in most other European countries, heroin addicts are institutionalized in opioid substitution therapy. The state‐of‐the‐art treatment replaces fast‐acting street opioids with slow‐acting ones, such as methadone. The primary objective of substitution therapy is harm reduction, by providing patients with stable doses of these drugs. In Austria, substitution is an outpatient therapy fully funded by statutory health insurance with low access barriers to substitution treatment, hence the majority of heroin users likely join the program eventually to secure a constant supply of opioids. This provides us with a close proxy for heroin addiction. Fourth and most importantly, we are able to link licit and illicit opioids use across generations with this near full‐coverage administrative data. Fifth, extensive information on the family environment allows us to test the degree to which the intergenerational persistence of opioid dependence is correlated with these and other known determinants of opioid addiction.

We find that if at least one parent uses illicit opioids, the likelihood that the child uses illicit opioids too increases from 1% to 7%. For prescription opioids, the intergenerational persistence estimate is slightly lower. It amounts to an increase from 3.6% to 6.7%. Both associations are precisely estimated (*p*‐value < 0.001).

Our results contribute to the literature in three main ways. *First* and foremost, we provide credible estimates of the intergenerational correlation of opioid dependence, with separate estimates for illicit and prescription opioids. While it is widely acknowledged in the literature on substance abuse that problems with addiction tend to run in families (Göhlmann et al., 2010; Henry & Augustyn, 2017; Kendler et al., 2015; Leonardi‐Bee et al., 2011; Mays et al., 2014; Melchior et al., 2010; Roettger et al., 2011; Schmidt & Tauchmann, 2011; Walters, 2002), very little is known about the case of opioids. A plausible explanation for this gap in the literature is the high data requirements. One not only needs to observe the consumption of prescription opioids or illicit opioids on an individual level, but also the ability to link this information across generations. The existing literature largely relies on small samples of addicts surveyed on their family background (Ellinwood et al., 1966; Hill et al., 1977; Maddux & Desmond, 1989; O'Donnell, 1969; Pohlisch, 1933).^3^ To our knowledge, the only exception is Log et al. (2013), who use the Norwegian Prescription Database covering the period 2004–2009 to obtain a sample of almost 100,000 Norwegian adolescents and their mothers. They find an association between maternal use of prescribed opioids and repeated use of their adolescent children. We are not aware of a large‐scale study on the intergenerational persistence of illicit opioid use. Our study is able to overcome the obstacles in the existing literature by leveraging administrative data comprising intergenerational links and using opioid substitution therapy as a proxy for illicit opioid use.

*Second*, since there is a close link between opioid dependence and mental health disorders (e.g., Davis et al., 2017; Halbert et al., 2016; Sullivan et al., 2006), our study also potentially speaks to the larger literature on the intergenerational transmission of health behavior (Thompson, 2014) and mental health (Johnston et al., 2013). There is perhaps also a connection to the intergenerational transmission of crime (Lindquist & Hjalmarsson, 2012; Williams & Sickles, 2002) and incarceration (Bhuller et al., 2018; Dobbie et al., 2018). Finally, to the extent that intergenerational persistence of opioid dependence is a hindrance to socioeconomic success, our results also speak to the broad literature on intergenerational mobility (Bowles & Gintis, 2002). Given the widespread and increasing misuse of opioids, our estimates of the intergenerational persistence in this domain are an important complement to the existing literature. These can help to improve our overall understanding of the factors that impede equality of opportunity, and to enhance our ability to remove those impediments in the area of mental health and resulting social problems.

*Third*, our results speak to the growing literature on the opioid crisis. An important strand of this literature discusses the causes of the US opioid crisis, and in particular whether it is supply or demand‐driven. So far, the literature has not reached a consensus. Case and Deaton (2017) attribute the surge in overdose deaths to worsening economic conditions and refer to the so‐called *deaths of despair* hypothesis. In contrast, Currie et al. (2018); Finkelstein et al. (2018); Hollingsworth et al. (2017); Ruhm (2018), and Schnell and Currie (2018) find little evidence for a causal impact of economic conditions and stress the importance of the availability of opioids.^4^ There are a number of studies evaluating interventions tackling the opioid epidemic, both on the supply‐side, such as prescription drug monitoring laws (Buchmueller & Carey, 2018; Gihleba et al., forthcoming; Grecu et al., 2019) or abuse‐deterrent drug formulations (Alpert et al., 2018; Evans et al., 2019), and on the demand‐side, such as syringe exchange programs (Packham, 2019). While we cannot speak to the relative importance of supply and demand‐side factors, our results suggest that any intervention which prevents or reduces opioid dependence today also has benefits for future generations.

The remainder of the paper is organized as follows. Section 2 summarizes the institutional setting with a focus on substitution therapy in Austria. Section 3 describes our data source and the definition of our estimation sample. Section 4 presents our estimation model. Section 5 discuss our estimates of the intergenerational correlation and shows how they change after to inclusion of covariates. Section 6 explores further alternative specifications, and Section 7 presents robustness checks. The final Section 8 concludes the paper.

## 2 INSTITUTIONAL SETTING

Opioid dependence is a complex health condition that requires long‐term treatment and medical care. The first‐line treatment recommended by the *World Health Organization* is medication‐assisted substitution therapy (WHO, 2009). In substitution programs, patients are prescribed specific opioids, such as buprenorphine, methadone, or morphine, which mimic the effects of heroin but are sufficiently long‐acting to avoid the cycles of intoxication and withdrawal. Programs have been shown to be effective in terms of substantially reducing illicit opiate use, HIV risk behaviors, death from overdose, criminal activity, and financial and other stresses on drug users and their families (Lawrinson et al., 2008). Although long‐term abstinence can be achieved and is sometimes desired, most patients are maintained on stable doses over time (Weigl & Busch, 2013).

In Austria, methadone has been used since the early 20th century. It had been prescribed as a last resort for long‐term addicts who had failed multiple withdrawal attempts in rehabilitation centers. Substitution therapy in its current form was established in 1998, when policy makers recognized it as being equally effective as abstinence treatment. The barrier to enter substitution therapy is low. Austria has a Bismarckian welfare system, which provides universal access to high‐quality healthcare for 99% of residents (Ahammer et al., 2021).^5^ Thus, every patient can enter substitution therapy practically for free. In principal, every patient who is diagnosed for opioid addiction and produces a positive urine screening on opioids will be admitted to the program. For patients under the age of 20, or when the patient declares to have taken opioids for less than 2 years, the prescribin general practionee (henceforth GP) has to consult with a psychiatrist to obtain a second opinion.

Treatment is primarily delivered by general practitioners. The most commonly used substitution drugs are methadone and buprenorphine. Every prescription has to be countersigned by the regional public health officer (PHO) before it can be dispensed daily and under supervision at a pharmacy. To minimize abuse, substitution prescriptions are only valid after the physician attaches a falsification‐proof authenticity sticker (in German “Vignette”) that contains a unique identification number. Those vignettes are recorded in an online system to which GPs, PHOs, and pharmacies have access to. This ensures that prescriptions cannot be forged and that patients can only obtain one prescription at a time. This is important, because there is less worry about diversion of substitution drugs to the black market, or multiple concurrent prescriptions through “doctor shopping.”

Since its introduction, the number of opioid users in substitution treatment has increased steadily. Official estimates suggest that 53% of opioid users were in treatment in 2019 (Horvath et al., 2019). However, this is likely only a lower bound of the true in‐treatment rate among opioid addicts. First, there is no reliable data on the number of opioid users in Austria, which makes it difficult to compute the denominator of the substitution prevalence rate with statistical certainty. Second, the denominator is based on an estimate of the number of all opioid addicts, not just regular ones.^6^ Third, the number represents a snapshot in time. A study from 2011 found that only 30% of patients currently in treatment had been on a stable dose for at least 12 months, the rest had multiple temporary interruptions (Weigl & Busch, 2013). If, despite these interruptions, at a given time as much as 55.3% of users are in substitution treatment, the lifetime prevalence is likely significantly higher. By observing addicts between 1998 and 2017, it is likely that we capture most of them at least once in our data. However, our results cannot speak to occasional opioid users, in that sense our sample may be negatively selected.^7^

Opioid prescriptions for pain are very similarly regulated as substitution prescriptions. They require special narcotic scripts, attached also with authenticity stickers. Thereby, every prescription is documented in an online monitoring system. Pain prescriptions are, however, subject to certain maximum amounts of the drug (e.g., 2 g morphine or 0.2 g oxycodone per patient). If the patient requires successive prescriptions for long‐term treatment, PHO approval becomes necessary, and every single prescription has to be countersigned before it can be dispensed at the pharmacy. Weak opioids, such as codeine or tramadol, are not subject to specific provisions when they are prescribed only once.

## 3 DATA AND SAMPLE DEFINITION

Our empirical analysis is based on linked data from several administrative registers. Most importantly, we have access to the *Upper Austrian Health Insurance* *Fund* database (henceforth UAHIF). This is the statutory health insurance provider that covers the population of all private‐sector workers and non‐employed residents in the province of Upper Austria.^8^ Importantly, also people receiving unemployment insurance or other social security benefits are insured with the UAHIF. This database includes detailed information on inpatient and outpatient healthcare expenditures. It also provides information on all prescribed medical drugs, which are coded using the *Anatomical Therapeutic Chemical Classification System* (ATC). This allows us to distinguish between opioids used in substitution therapy and those used to treat pain. Substances that are used in either therapy, however, carry a different ATC code depending on their purpose.^9^ Below we use the term “prescription opioids” to refer to opioids used in pain therapy. In particular, these are all drugs in ATC categories N01AH and N02A that require a prescription in Austria.

**FIGURE 1:**
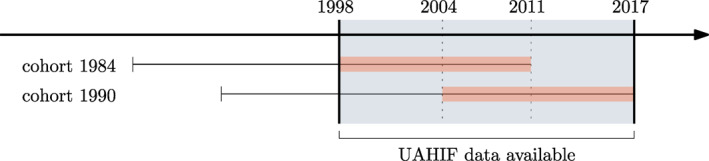
Birth cohorts in study and data availability. This figure illustrates the birth cohorts in study and data availability. UAHIF health records are available between 1998 and 2017, this is the blue shaded area. We consider the cohorts 1984–1990, where we observe each child from age 14 to 27. For example, a child born in 1984 is 14 in 1998 and 27 in 2011. A child born in 1990 is 14 in 2004 and 27 in 2017. Our results are robust to choosing different cohorts; in Figure A2, we extend the window to children born between 1980 and 1990, which leads to very similar conclusions as in our baseline. UAHIF, Upper Austrian Health Insurance Fund

**FIGURE 2:**
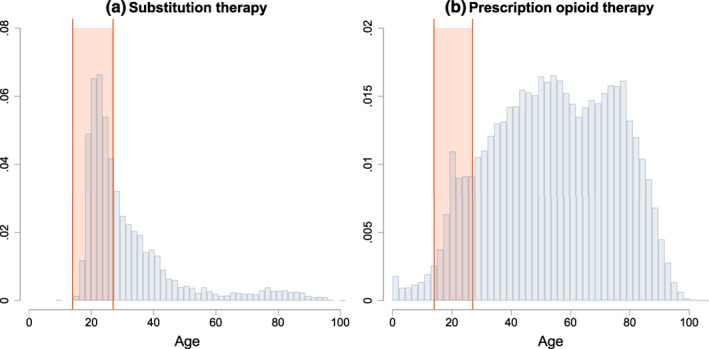
Age at onset of substitution and prescription opioid therapy. This figure shows distributions of the age at onset for the first recorded (a) substitution therapy and (b) prescription opioid therapy per patient in the full 1998–2017 UAHIF database. The shaded area indicates the age range 14–27, which is the period in which we observe substitution therapy and prescription opioid therapy for the children in our sample. UAHIF, Upper Austrian Health Insurance Fund

We examine children born between 1984 and 1990. Since we have birth record data for 1998–2017, this choice of birth cohorts allows us to observe children between 14 and 27 years of age (see Figure 1).^10^ This is the age range where most patients initiate substitution therapy in Upper Austria. In Figure 2, we plot distributions of the age at onset for substitution and prescription opioid therapy, based on the full 1998–2017 UAHIF sample. The shaded area highlights the age range 14–27. The pattern in Panel (a) is consistent with survey evidence suggesting that illicit opioid use peaks between 18 and 25 years of age (Hu et al., 2017).

Information on parent‐child pairs comes from official records and is typically measured at birth. Information on the link between mothers and children is complete. The link between fathers and children is missing in 11.3% of cases.^11^ After linking children to parents, we obtain an estimation sample comprising 81,307 child‐parent pairs.^12^ Table A1 provides key descriptive statistics. Among these children, about 1.1% had been in substitution therapy at some point in time. Their average age at onset is 22 years and less than 0.2% of children in the substitution program had started therapy before the beginning of the sample period (i.e., are left censored). Since, presumably, it is rare that children started *and* ended opioid abuse before the age of 14, we are confident that our data allow us to capture children's lifetime prevalence in illicit opioid use.

The median birth years of mothers and fathers are 1961 and 1959, respectively. Thus, for the median mother, we observe opioid usage starting from the 37th birthday. The addiction prevalence rates among parents are comparably lower. They amount to 0.3% for mothers and 0.5% for fathers. This generational gap can be explained a difference in the lifetime prevalence of heroin use across birth cohorts. This is consistent with Giordano et al. (2014), who find that Swedes born in the 1980s and 1990s have significantly higher hospitalization rates for drug abuse than those born in the 1960s and 1970s. Alternatively, it could reflect measurement error. It is possible that parents had been in substitution therapy before we observe them in our sample (in 1998), but have not enrolled anymore thereafter. While this timing certainly does not represent a typical pattern, it is worth noting that the resulting measurement error would lead to an attenuation bias.^13^ Thus, we would obtain smaller, more conservative intergenerational persistence estimates for heroin use.

The incidence of prescription opioid use is lower for children (4.6%) than for their parents (mothers: 20.4%, fathers: 17.7%). An important factor in explaining this difference across generations is certainly age. Since we have seen before that prescription opioid use peaks later in life, we can therefore not claim to measure lifetime prevalence in prescription opioid use for children. For our main analysis, we define parental opioid use as either the mother or the father being in substitution therapy or being prescribed prescription opioids. According to these definitions, we observe a parental life time prevalence of heroin use of 0.6% and of prescription opioids use of 33.3%.

## 4 METHODOLOGY

To examine the intergenerational link in opioid use in family *j*, we relate an indicator variable for child opioid use, $a_{j}^{c}$, to an indicator variable for parent opioid use, $a_{j}^{p}$,

(1) $$a_{j}^{c}=\alpha \cdot a_{j}^{p}+\beta^{c}B_{j}^{c}+\beta^{p}B_{j}^{p}+\gamma E_{j}+\eta_{j},$$

where the superscripts *c* and *p* denote children and parents, respectively. We run separate ordinary least squares (OLS) regressions for illicit opioids and prescription opioid use. For both measures, the parameter of primary interest is *α*, which captures the intergenerational persistence of opioid dependence. This is an overall measure of the parent‐child correlation in opioid use, which may comprise both the genetic transmission of parental characteristics (“nature”), the environment a child is growing in (“nurture”), or a combination of both. While this distinction is important, a separation from nature and nurture is beyond the scope of this paper. However, we present different specifications below where we control for a wide range of socioeconomic and environmental factors.^14^ One caveat is that we cannot say anything about drugs other than opioids. We distinguish between three sets of controls; the child's conditions at birth and socioeconomics, $B_{j}^{c}$, the mother's socioeconomic characteristics, $B_{j}^{p}$, and environmental factors outside the family, *E*_*j*_.

We also provide specifications where we interact different sets of mother socioeconomics (i.e., age at birth, $age_{j}^{p}$, born in wedlock, $wedlock_{j}^{p}$, education, $educ_{j}^{p}$, and job, $job_{j}^{p}$) with our measure of illicit opioid use, first separately and then together in one regression:

(2) $$a_{j}^{c}=\alpha \cdot a_{j}^{p}+\zeta_{1}age_{j}^{p}+\delta_{1}\left(a_{j}^{p}\cdot age_{j}^{p}\right)+\eta_{j}$$

(3) $$a_{j}^{c}=\alpha \cdot a_{j}^{p}+\zeta_{2}wedlock_{j}^{p}+\delta_{2}\left(a_{j}^{p}\cdot wedlock_{j}^{p}\right)+\eta_{j}$$

(4) $$a_{j}^{c}=\alpha \cdot a_{j}^{p}+\zeta_{3}educ_{j}^{p}+\delta_{3}\left(a_{j}^{p}\cdot educ_{j}^{p}\right)+\eta_{j}$$

(5) $$a_{j}^{c}=\alpha \cdot a_{j}^{p}+\zeta_{4}job_{j}^{p}+\delta_{4}\left(a_{j}^{p}\cdot job_{j}^{p}\right)+\eta_{j}$$

(6) $$\begin{array}{ccc} a_{j}^{c} & = & \alpha \cdot a_{j}^{p}+\zeta_{1}age_{j}^{p}+\zeta_{2}wedlock_{j}^{p}+\zeta_{3}educ_{j}^{p}+\zeta_{4}job_{j}^{p}\\ & + & \delta_{1}\left(a_{j}^{p}\cdot age_{j}^{p}\right)+\delta_{2}\left(a_{j}^{p}\cdot wedlock_{j}^{p}\right)+\delta_{3}\left(a_{j}^{p}\cdot educ_{j}^{p}\right)+\delta_{4}\left(a_{j}^{p}\cdot job_{j}^{p}\right)+\eta_{j}, \end{array}$$

where *δ*_1_, …, *δ*_4_ are the coefficients on the interaction terms. These terms measure how each covariate increases or decreases the intergenerational persistence of opioid use. In all our specifications we cluster standard errors on the municipality level. Clustering on the family level provides unchanged results (see Table A2).

## 5 RESULTS

**FIGURE 3:**
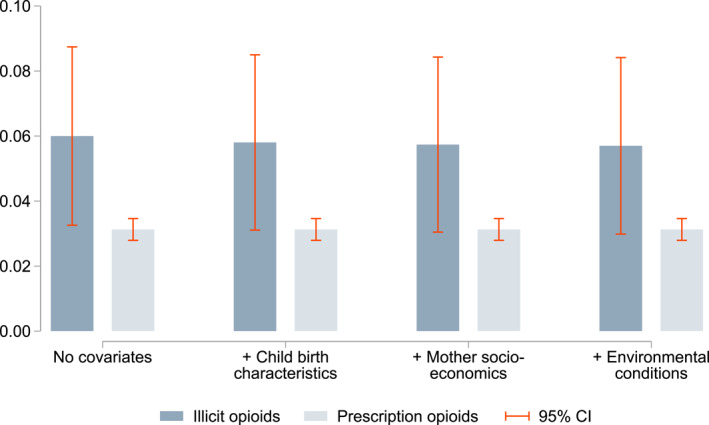
The intergenerational persistence of opioid dependence. This graph plots the intergenerational persistence estimates for illicit opioids and prescription opioids with varying covariates. Dependence to illicit opioids is approximated with participation in substitution therapy. Prescription opioids comprise all drugs in ATC categories N01AH and N02A. Children are considered to exposed to parental opioid use if either the father or the mother have ever been using illicit opioids or prescription opioids, respectively. The bars represent OLS estimates and 95% confidence intervals based on municipality‐level clustered and heteroskedasticity‐robust standard errors are indicated by the purple lines. The covariate included are listed in Table 1. ATC, Anatomical Therapeutic Chemical Classification System

Our estimation results are summarized in Figure 3. Full estimation outputs are listed in Tables 1 (illicit opioids) and 2 (prescription opioids). We find clear evidence of an intergenerational persistence of opioid use. Our unconditional estimate for illicit opioid abuse of 0.06 indicates that a heroin user's child is 6.0% points more likely to use heroin themselves compared to the child of non‐using parents. Put differently, if at least one parent is using heroin, the likelihood of the child being addicted increases from 1% to 7% (or 7 times more likely). The intergenerational persistence estimate for prescription opioids is about half and amounts to 0.03. Thus, if at least one parent uses prescription opioids, the likelihood of the child using increases from 3.6% to 6.7%. Both coefficients are precisely estimated (*p*‐value < 0.001) and remarkably stable when introducing socioeconomic covariates. These two estimates cannot be interpreted as structural parameters of the intergenerational transmission process. Instead, they are catch‐all measures of the intergenerational association that encompass a variety of transmission mechanisms.

**TABLE 1:** The intergenerational persistence of opioid dependence: Illicit opioids

	(1)	(2)	(3)	(4)	(5)
Mother or father have been in opioid substitution	0.060*** (0.014)	0.058*** (0.014)	0.057*** (0.014)	0.057*** (0.014)	
Child birth conditions and socioeconomics					
Birth weight below 2500 g		−0.004* (0.002)	−0.003* (0.002)	−0.003 (0.002)	−0.003 (0.002)
ln (length at birth)		−0.010 (0.009)	−0.005 (0.008)	−0.002 (0.008)	−0.003 (0.008)
Female		−0.008*** (0.001)	−0.008*** (0.001)	−0.008*** (0.001)	−0.008*** (0.001)
Born in wedlock		−0.008*** (0.001)	−0.007*** (0.001)	−0.006*** (0.001)	−0.006*** (0.001)
Urban region		0.011** (0.005)	0.011** (0.005)	0.003 (0.004)	0.003 (0.004)
Mother socioeconomics					
Age at birth (years/10)			−0.002 (0.002)	−0.004 (0.002)	−0.004 (0.002)
Age in 1998 (years/10)			−0.002 (0.002)	−0.001 (0.003)	−0.001 (0.003)
Religion (baseline: Catholic)					
Protestant			0.005*** (0.002)	0.003* (0.002)	0.003* (0.002)
Muslim			0.024*** (0.006)	0.021*** (0.006)	0.021*** (0.006)
Other			0.008*** (0.002)	0.006*** (0.002)	0.007*** (0.002)
Job (baseline: white collar worker)					
Entrepreneur or freelancer			−0.002 (0.002)	−0.001 (0.002)	−0.001 (0.002)
Housewife			−0.002 (0.001)	−0.001 (0.001)	−0.001 (0.001)
Blue collar worker			0.005*** (0.001)	0.005*** (0.001)	0.005*** (0.001)
Education (baseline: compulsory education)^a^					
Apprenticeship training			−0.001 (0.001)	−0.002 (0.001)	−0.002 (0.001)
High school (without A‐levels)			−0.002* (0.001)	−0.002** (0.001)	−0.002*** (0.001)
High school			−0.006*** (0.002)	−0.006*** (0.001)	−0.006*** (0.001)
Vocational school			−0.006*** (0.001)	−0.007*** (0.001)	−0.007*** (0.001)
University			−0.004 (0.003)	−0.005* (0.003)	−0.006* (0.003)
Environmental conditions at age 14^b^					
No. of substituting GPs in district per 1000 population				0.008 (0.008)	0.008 (0.008)
Share of population employed				−0.059* (0.031)	−0.058* (0.031)
Ethnic composition of municipality					
Share of former Yugoslavs				0.169*** (0.027)	0.170*** (0.027)
Share of Turks				0.045 (0.051)	0.046 (0.051)
Share of Germans				−0.039 (0.026)	−0.041 (0.026)
Share of other immigrants				−0.085 (0.090)	−0.079 (0.089)
Intercept	0.010*** (0.001)	0.059* (0.034)	0.051 (0.034)	0.058 (0.036)	0.064* (0.036)
*F*‐statistic	18.4	20.7	13.7	15.4	14.5
Adjusted *R* ^2^	0.002	0.007	0.009	0.011	0.009
Number of observations	81,307	81,307	81,307	81,307	81,307
Number of children substituted	869	869	869	869	869
Number of parents substituted	512	512	512	512	512

*Note*: This table presents regression results for the determinants of a child being in the opioid substitution therapy before the age of 27. The sample is based on the universe of children born between 1984 and 1990. Municipality‐level clustered standard errors given in parentheses, stars indicate statistical significance: * *p* < 0.10, ** *p* < 0.05, *** *p* < 0.01.

^a^ In Austria there are 9 years of compulsory education.

^b^ Variables capturing the environmental condition are measured at the municipality level. The only exception is GP density, which is measured on the district level.

**TABLE 2:** The intergenerational persistence of opioid dependence: Prescription opioids

	(1)	(2)	(3)	(4)	(5)
Mother or father have used prescription opioid	0.031*** (0.002)	0.031*** (0.002)	0.028*** (0.002)	0.027*** (0.002)	
Child birth conditions and socioeconomics					
Birth weight below 2500 g		−0.007 (0.004)	−0.006 (0.004)	−0.006 (0.004)	−0.005 (0.004)
ln (length at birth)		−0.032 (0.021)	−0.025 (0.021)	−0.017 (0.021)	−0.022 (0.021)
Female		0.002 (0.001)	0.002 (0.001)	0.002 (0.001)	0.002 (0.001)
Born in wedlock		−0.012*** (0.002)	−0.009*** (0.002)	−0.008*** (0.002)	−0.008*** (0.002)
Urban region		0.014*** (0.004)	0.015*** (0.003)	−0.006 (0.005)	−0.008 (0.005)
Mother socioeconomics					
Age at birth (years/10)			−0.021*** (0.004)	−0.030*** (0.004)	−0.033*** (0.004)
Age in 1998 (years/10)			0.015*** (0.003)	0.025*** (0.004)	0.029*** (0.004)
Religion (baseline: Catholic)					
Protestant			0.003 (0.004)	−0.000 (0.004)	0.001 (0.005)
Muslim			0.027*** (0.007)	0.023*** (0.007)	0.030*** (0.007)
Other			0.015** (0.007)	0.013** (0.006)	0.014** (0.006)
Job (baseline: white collar worker)					
Entrepreneur or freelancer			0.007 (0.006)	0.009 (0.006)	0.008 (0.006)
Housewife			−0.007** (0.003)	−0.004 (0.003)	−0.008** (0.003)
Blue collar worker			0.007*** (0.002)	0.007*** (0.002)	0.008*** (0.002)
Education (baseline: compulsory education)^a^					
Apprenticeship training			−0.005*** (0.002)	−0.006*** (0.002)	−0.007*** (0.002)
High school (without A‐levels)			−0.010*** (0.002)	−0.011*** (0.002)	−0.014*** (0.002)
High school			−0.014*** (0.003)	−0.016*** (0.003)	−0.020*** (0.003)
Vocational school			−0.021*** (0.003)	−0.023*** (0.004)	−0.029*** (0.004)
University			−0.016*** (0.006)	−0.017*** (0.006)	−0.023*** (0.006)
Environmental conditions at age 14^b^					
No. of substituting GPs in district per 1000 population				0.056*** (0.016)	0.065*** (0.017)
Share of population employed				−0.114*** (0.041)	−0.114*** (0.042)
Ethnic composition of municipality					
Share of former Yugoslavs				0.210*** (0.041)	0.227*** (0.042)
Share of Turks				0.101 (0.070)	0.117 (0.071)
Share of Germans				−0.069 (0.058)	−0.080 (0.060)
Share of other immigrants				−0.099 (0.108)	−0.086 (0.110)
Intercept	0.036*** (0.001)	0.170** (0.083)	0.136 (0.085)	0.137 (0.089)	0.154* (0.089)
*F*‐statistic	335.4	64.9	31.3	32.7	23.7
Adjusted *R* ^2^	0.005	0.006	0.008	0.009	0.006
Number of observations	81,307	81,307	81,307	81,307	81,307
Number of children taking pain drugs	3770	3770	3770	3770	3770
Number of parents taking pain drugs	27,053	27,053	27,053	27,053	27,053

*Note*: This table presents regression results for the determinants of a child using prescription opioids before the age of 27. The sample is based on the universe of children born between 1984 and 1990. Municipality‐level clustered standard errors given in parentheses, stars indicate statistical significance: * *p* < 0.10, ** *p* < 0.05, *** *p* < 0.01.

^a^ In Austria there are 9 years of compulsory education.

^b^ Variables capturing the environmental condition are measured at the municipality level. The only exception is GP density, which is measured on the district level.

To compare our estimate to the existing literature, it is useful to compute odds ratios. Using a logit model, we obtain odds ratios of 5.78 for illicit opioids and 1.76 for prescription opioids after controlling for child birth characteristics, mother socioeconomics, and environmental conditions (see Table A3). This is well within the range of intergenerational persistence estimates reported for other drugs. For example, Henry and Augustyn (2017) find an intergenerational odds ratio for cannabis use of 9.70, but their confidence interval (3.00, 31.34) encompasses our estimate for illicit opioids. The intergenerational correlation of licit drug abuse appears to be weaker. Kendler et al. (2015) find an odds ratio of 1.46 for the effect of parental alcohol use disorder on children being diagnosed with the same. Leonardi‐Bee et al. (2011), in a meta‐study, find a similar odds ratio for smoking at 1.72. This is very close to our estimate for the intergenerational persistence of licit opioids. Using a composite measure of substance abuse (including both licit and illicit drugs), Thornberry et al. (2006) report an intergenerational odds ratio of 2.1, which is again in the neighborhood of our estimates.

While we do not aim to quantify the relative contribution from nature (inherited genes) and nurture (upbringing), it is still instructive to examine how the intergenerational persistence estimate is affected when including different types of covariates. We distinguish between three broad categories. First, we include information on the child's condition at birth, controlling for sex, birth weight, birth length, whether the child was born in wedlock, and place of residence. Second, we introduce measures for the mother's socioeconomic status at the time of birth. In particular, we control for the mother's age, religious denomination,^15^ educational attainment, and occupation. Third, we account for environmental factors outside the family. Here we aim to capture (or at least proxy for) neighborhood conditions and the local supply of opioids that the previous literature suggests is correlated with use (e.g., Fuller et al., 2005) and drug‐related policing (e.g., Beckett et al., 2006). To capture the local economic situation and demographic environment, we control for the share of population in employment and the ethnic composition of the municipality. To approximate the local supply of opioids we use the number of GPs in the district providing substitution therapy. The idea here is that there is a correlation between the number of GPs offering opioid substitution therapy and the number of opioid users in a community. The inclusion of this large set of covariates does not alter the estimated intergenerational persistence of opioid usage at all. A comparison of the bars in Figure 3 shows that this intriguing finding holds for both illicit and prescription opioids. Detailed estimation output is available in columns (2) to (4) of Tables 1 and 2. In the following we discuss signs and magnitudes of selected control variable coefficients.

*Child birth conditions and socioeconomics*: Better birth conditions are associated with a lower probability of being an opioid user, but become insignificant once environmental conditions controlled for (column 4). We see this effect only for illicit opioids, for prescription opioids birth conditions are not significant. Children born in wedlock are significantly less likely to use illicit opioids and to take prescription opioids. Sex is only relevant for the former but not the latter.

*Mother socioeconomics*: Mother socioeconomics have large effects on child opioid use, even in the full model controlling for a variety of other determinants. Children of Catholic mothers have the smallest probability to use opioids, children of Muslims the highest, controlling for socioeconomic characteristics.^16^ Furthermore, children of blue collar workers are more likely to use opioids, while better education is a protective factor. Maternal age is significant only for prescription opioids, with age at birth being negatively and age in 1998 being positively correlated with child use.

*Environmental conditions*: While our proxy for opioid supply, the density of substituting GPs in the district, is insignificant, we find that neighborhood conditions are important. The former is surprising, but perhaps a result of the other variables in this category picking up variation in opioid supply too. We see that, the higher employment in the community, the lower the child's chance to use opioids. The ethnic composition is an important predictor too, with coefficients being jointly significant at the 1% level for both illicit (*F* = 16.4) and licit opioid use (*F* = 8.8). Note that this does not mean that ethnicity per se has an impact on drug use; the ethnic composition may simply be correlated with other neighborhood characteristics that do so.

In sum, we see that inclusion of covariates capturing the child's environment inside and outside the family (such as child's birth weight or the local employment rate) does not alter our persistence estimates.

## 6 ALTERNATIVE SPECIFICATIONS

In this section, we explore further alternative specifications. We first ask whether there are observable factors that affect the strength of the intergenerational persistence. In a second step, we explore whether the intensity to exposure matters for the strength of intergenerational persistence.

### 6.1 Moderating observable factors

To explore whether there are observable factors that protect (or promote) intergenerational persistence, we interact the mother's education, job, age at birth, and whether the child is born in wedlock with our indicator for parental heroin dependence, as shown in Equations (2), (3), (4), (5). This sheds light on whether there are factors that make persistence more or less likely. Table 3 reports the coefficients on these interaction terms. When introduced separately, we find that the interactions with being born in wedlock and certain educational and job characteristics are significant. While age at birth does not affect the intergenerational persistence of opioid use (column 1); if the child is born in wedlock (column 2), if the mother has vocational or university education (column 3), and if the mother is a housewife or a freelancer (column 4) all reduce the persistence estimate. In column (5), we add all these interaction terms together in one model, as in Equation (6). Here we see that being born in wedlock reduces the intergenerational persistence by 8.4% points, and being a freelancer or a housewife reduces the persistence by 6.7 and 5% points, respectively. Education is not significant anymore. This suggests that having a stay‐home mother and being born into an ongoing marriage are strong protective factors.

**TABLE 3:** Interacting parental opioid use with important socio‐economic status characteristics

	(1)	(2)	(3)	(4)	(5)
Mother or father have been in opioid substitution	0.091* (0.054)	0.119*** (0.031)	0.052*** (0.018)	0.066*** (0.017)	0.094* (0.054)
× Age at birth	−0.012 (0.019)				0.005 (0.021)
× Born in wedlock		−0.083*** (0.031)			−0.084*** (0.032)
Education (baseline: compulsory education)^a^					
× Apprenticeship training			0.005 (0.035)		0.011 (0.043)
× High school (without A‐levels)			0.050 (0.047)		0.054 (0.056)
× High school			−0.016 (0.047)		0.000 (0.053)
× Vocational school			−0.054*** (0.018)		−0.028 (0.039)
× University			−0.058*** (0.019)		−0.032 (0.040)
Job (baseline: white collar worker)					
× Entrepreneur or freelancer				−0.070*** (0.017)	−0.067** (0.032)
× Housewife				−0.069*** (0.017)	−0.050*** (0.019)
× Blue collar worker				−0.003 (0.020)	0.000 (0.025)
Age at birth	Yes	No	No	No	Yes
Born in wedlock	No	Yes	No	No	Yes
Education	No	No	Yes	No	Yes
Job	No	No	No	Yes	Yes
*F*‐statistic for interaction term (s)	0.41	7.31	7.58	7.16	3.61
*p*‐value for interaction term (s)	0.520	0.007	0.000	0.000	0.000

*Note*: This table estimates for models in which we interact parental heroin use with age at birth (column 1), an indicator for whether the child is born in wedlock (column 2), the mother's education (column 3) and job (column 4), and all these variables together (column 5). The models are fully interacted, but we only report the coefficient on parental heroin use and the coefficients on the interaction terms. The former has to be interpreted as the intergenerational persistence estimate when all interacted covariates are 0. The coefficients on the interaction terms can be interpreted as the differences in intergenerational persistence by the interacted variable. The *F*‐statistics test for the joint significance of the interaction terms in each model. Municipality‐level clustered standard errors given in parentheses, stars indicate statistical significance: * *p* < 0.10, ** *p* < 0.05, *** *p* < 0.01.

^a^ In Austria there are 9 years of compulsory education.

### 6.2 Exposure intensity

Now we exploit information on the timing and duration of parental opioid use. To do so, we construct three different measures for both substitution and prescription opioid therapy: (i) Total prescription days: We count the total number of days either parent was in substitution or prescription opioid therapy (conditional on being in therapy at least once between 1998 and 2017). (ii) “On‐&‐off”: To identify parents that switch into and out off substitution and prescription opioid therapy, we define a binary variable indicating whether at least one parent has repeated substitution/prescription opioid therapy spells between 1998 and 2017. (iii) “Already in 1998”: We define a binary variable which captures whether therapy for either parent is left‐censored. That is, whether there was already a substitution/opioid prescription in 1998. This is a proxy for whether parents had already been in therapy before we start observing them in our data.

For each of these measures, we assign opioid‐using parents to one of two groups: (i) Parents with below or at median prescription days versus above median prescription days, (ii) parents with a single spell versus repeated spells, (iii) parents already in therapy in 1998 versus those that joined later. We then estimate regressions where our main explanatory variable—exposure to parental opioid addiction—is only one if parents are in the respective group, and drop parents that are in the other group from the sample. The estimated correlations are relative to children with parents that do *not* use opioids at all; this group appears in all regressions.

Table 4 summarizes our estimation results. As expected, the intergenerational persistence estimates are slightly stronger when children are exposed to more and longer opioid use. For example, if children are only considered exposed if parents have above median substitution medication prescription days (panel a, column 2), the estimate is 7.3% points, while it is 4.7% points when exposure is defined as parents having below median prescription days (panel a, column 2). However, when we test for the equality of these coefficients, we cannot reject the hypothesis that they are the same (*p* = 0.151). The same holds true when defining exposure according to on‐&‐off consumption patterns or whether parents had already been using in 1998, where tests for the equality of coefficients give *p* = 0.554 and 0.963, respectively.

**TABLE 4:** Different parental opioid use measures

	Total days		On/off		Already in 1998	
	≤ Median	> Median	No	Yes	No	Yes
	(1)	(2)	(3)	(4)	(5)	(6)
(a) Illicit opioids						
Intergenerational persistence	0.047*** (0.015)	0.073*** (0.019)	0.055*** (0.014)	0.068*** (0.023)	0.057*** (0.013)	0.058** (0.026)
Child birth characteristics	Yes	Yes	Yes	Yes	Yes	Yes
Mother socioeconomics	Yes	Yes	Yes	Yes	Yes	Yes
Environmental conditions	Yes	Yes	Yes	Yes	Yes	Yes
*χ* ^2^ Test for equality of coefficients	2.062 (*p* = 0.151)		0.350 (*p* = 0.554)		0.002 (*p* = 0.963)	
Number of observations	81,099	81,003	81,207	80,895	81,209	80,893
(b) Prescription opioids						
Intergenerational persistence	0.019*** (0.002)	0.047*** (0.003)	0.019*** (0.002)	0.046*** (0.003)	0.029*** (0.002)	0.040*** (0.005)
Child birth characteristics	Yes	Yes	Yes	Yes	Yes	Yes
Mother socioeconomics	Yes	Yes	Yes	Yes	Yes	Yes
Environmental conditions	Yes	Yes	Yes	Yes	Yes	Yes
*χ* ^2^ Test for equality of coefficients	76.657 (*p* = 0.000)		74.590 (*p* = 0.000)		4.082 (*p* = 0.043)	
Number of observations	70,493	65,068	69,700	65,861	77,957	57,604

*Note*: This table presents regression results for the determinants of a child using illicit opioids (panel a) and prescription opioids (panel b) before the age of 27. In columns (1), we only consider opioid‐using parents when the total number of days either of the parents is prescribed an opioid between 1998 and 2017, conditional on being prescribed an opioid at least once, is smaller than or at the sample median (213 days for illicit opioids and 30 days for prescription opioids), and drop parents from the sample whose total number of prescription days is smaller than the median. In column (2) we consider only parents for whom the total number of days either of the parents is prescribed an opioid is larger than the sample median, and drop parents from the sample whose total number of prescription days is larger than the median. In columns (3) and (4), we set parental opioid use to one if both parents have at most one prescription spell (dropping parents with multiple spells, column 3), or multiple spells with at least 90 days in‐between (dropping parents with only one spell, column 4). In columns (5) and (6), we set parental opioid use to one if either of the parents had already been prescribed an opioid when the sample starts in 1998 (dropping parents whose first prescription was after 1998, column 5), or if both parents first prescription was in or after 1999 (dropping all parents with prescriptions before 1999, column 6). The sample is based on the universe of children born between 1984 and 1990. Stars indicate statistical significance: * *p* < 0.10, ** *p* < 0.05, *** *p* < 0.01. The *χ* ^2^ tests for the equality of coefficients are from seemingly unrelated regressions with two equations each (columns 1 and 2, 3 and 4, 5 and 6).

For prescription opioids, we do see that more exposure (columns 2, 4, and 6) leads to significantly larger estimates, which are much closer to the illicit opioid estimates in our baseline estimation. This is perhaps unsurprising, as longer and repeated prescription opioid spells hint at actual addiction, while single and shorter spells (columns 1, 3, and 5) may catch parents that were “properly” using opioids for health problems and had not become addicted. This can explain the drop in coefficients from 0.057 to 0.027 between substitution therapy and prescription opioid use, because the latter partly captures also non‐addicted parents.

## 7 ROBUSTNESS CHECKS

In this section, we examine the robustness of our results. First, we try different definitions of the our main variable capturing exposure to parental opioid use. Second, we vary the birth cohorts of children included in our analysis.

### 7.1 Definition of exposure to parental opioid use

**FIGURE 4:**
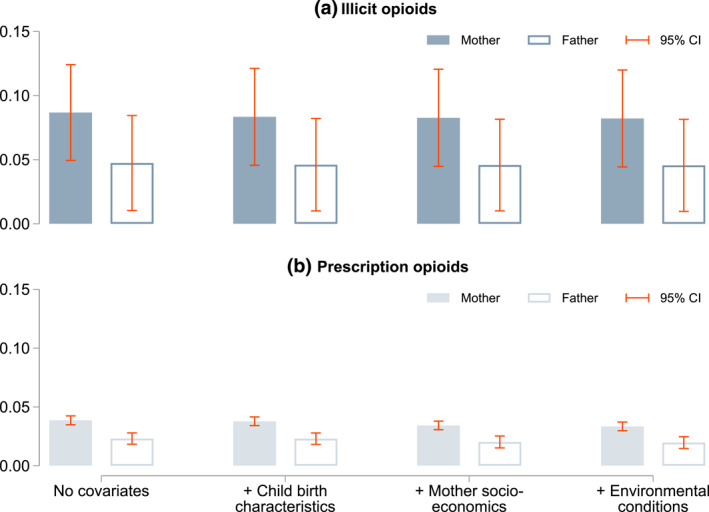
The intergenerational persistence of opioid dependence: Effect of mothers versus fathers. This graph plots the intergenerational persistence estimates for (a) illicit opioids and (b) prescription opioids with varying covariates. Dependence to illicit opioids is approximated by participation in substitution therapy. We present results based on two treatment definitions. First, a child is considered exposed to parental opioid use if their mother has ever been in substitution therapy (filled bars). These estimations are based on a sample of 74,909 mother‐child pairs, containing 225 substituted mothers and 844 substituted children. Second, a child is considered exposed to parental opioid use if their father has ever been in substitution therapy (hollow bars). These estimations are based on a sample of 60,649 father‐child pairs, which comprises 301 substituted fathers and 578 substituted children. Prescription opioids comprise all drugs in ATC categories N01AH and N02A. Here the mother‐child pairs (filled bars) contain 16,345 mothers and 3583 children with at least one prescription. The father‐child pairs (hollow bars) contain 14,364 fathers and 2698 children. The covariates included are listed in Table 1. The bars represent OLS estimates and 95% confidence intervals based on municipality‐level clustered and heteroskedasticity‐robust standard errors are indicated by the orange lines. ATC, Anatomical Therapeutic Chemical Classification System

In Panel (a) of Figure 4, we present results for the intergenerational persistence of illicit opioids based on two alternative definitions of exposure to parental opioid use. First, a child is considered exposed if the mother has ever been in substitution therapy (see the filled bars). These estimations are based on a sample of 74,909 mother‐child pairs, comprising 225 substituted mothers and 844 substituted children. Second, a child is considered exposed if the father has ever been in substitution therapy (see hollow bars). These estimations are based on a sample of 60,649 father‐child pairs, comprising 301 substituted fathers and 578 substituted children. Panel (b) depicts equivalent results for prescription opioids. Here the mother‐child pairs (filled bars) contain 16,345 mothers and 3583 children with at least one prescription. The father‐child pairs (hollow bars) contain 14,364 fathers and 2698 children. Details are provided in the notes to Figure 4.

Across outcomes and specifications we find significant estimates of the intergenerational persistence of opioid dependence. Point estimates are consistently larger for the exposure based on maternal opioid use, but 95% confidence intervals are overlapping for illicit opioids. For prescription opioids, the differences are quantitatively negligible. Furthermore, since estimates comparing maternal and paternal opioid use are not directly comparable due differences in sample composition (in particular, we do not observe single fathers in our data), these results do not necessarily suggest that mothers are more likely to transmit opioid use.

### 7.2 Birth cohorts considered

**FIGURE A2:**
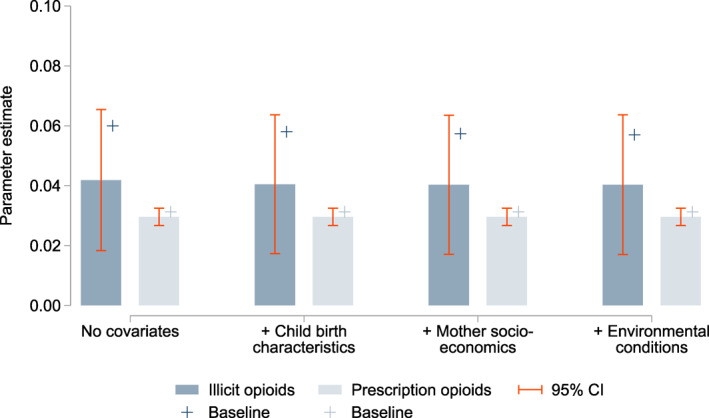
Intergenerational persistence of opioid dependence, extended sample 1980–1990. This graph replicates the regressions in Figure 3 on a sample that also includes children born between 1980 and 1983. For these cohorts we do not have information on mothers' education and job, we therefore do not control for these variables when we introduce mother socioeconomics. The bars represent OLS estimates and 95% confidence intervals based on municipality‐level clustered and heteroskedasticity‐robust standard errors are indicated by the purple lines. The covariates included are listed in Table 1

**FIGURE A3:**
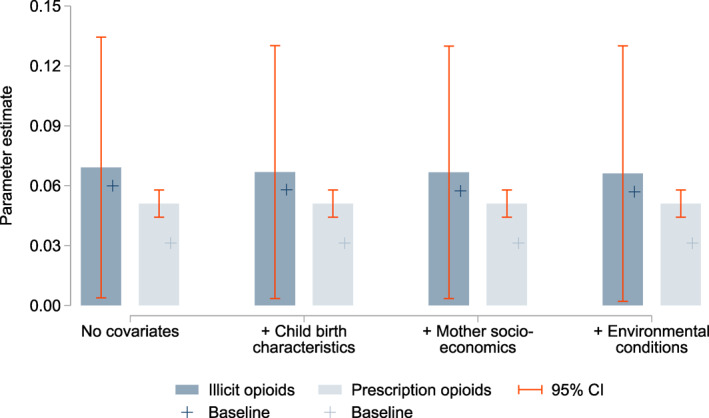
Intergenerational persistence of opioid dependence, restricted sample 1984–1985. This graph replicates the regressions in Figure 1 on a sample that also includes only children born between 1984 and 1985. This allows us to observe children between ages 14–32. The bars represent OLS estimates and 95% confidence intervals based on municipality‐level clustered and heteroskedasticity‐robust standard errors are indicated by the purple lines. The covariate included are listed in Table 1

Finally, we show that our estimation results do not depend on the cohorts of children chosen. In our baseline estimates, we focus on children born between 1984 and 1990. This allows us to observe children during adolescence and early adulthood (i.e., between the age of 14–27). Figure A2 replicates our estimation for children born between 1980 and 1990. This results in a larger sample size, but prevents us from observing children younger than 18. Additionally, we do not have information on mothers' education and job at birth, because these variables are not available in the birth register prior to 1984. The resulting point estimates for illicit opioids are somewhat smaller, but the 95% confidence intervals cover our baseline estimate. The estimates for prescription opioids are essentially unchanged. As a second exercise, we omit the last five cohorts in our sample in order to observe children 5 years longer into their adulthood; that is, until the age of 32. This reduces the sample to two cohorts (1984 and 1985). Results are shown in Figure A3. While the estimates for illicit opioids are again similar to the baseline, the estimates for prescription opioids increase by roughly 1% point.

## 8 CONCLUSIONS

This is the first paper to provide credible estimates of the intergenerational persistence of licit and illicit opioid usage. Using administrative data sources from Austria, we show that the usage of heroin and prescription opioids are both strongly correlated within families. We find that, if at least one parent is using illicit opioids, the likelihood of the child using increases from 1% to 7%. For prescription opioids, we observe an increase from 3.6% to 6.7%. These estimates are well within the range of intergenerational persistence estimates reported for other drugs, such as alcohol (Kendler et al., 2015), tobacco (Leonardi‐Bee et al., 2011), and cannabis (Henry & Augustyn, 2017).

While we do not aim to quantify the relative contribution from nature (inherited genes) and nurture (upbringing), we demonstrate that both associations are stable and do not change when controlling for environmental variables. An obvious caveat is that we cannot control for the underlying cause of opioid use persistence across generations. The next step for research is to unpack the different mechanisms of transmission by which parental addiction affects child opioid use. In terms of the ongoing opioid crisis in the US and Canada, there is the concern that the surge in opioid dependence has negative spillovers to the children of today's users. Thus, any intervention which prevents or reduces opioid dependence today may also have benefits for future generations.

## CONFLICT OF INTEREST

The authors declare no conflict of interest.

## Data Availability

The data that support the findings of this study are available from the Upper Austrian Health Insurance Fund. Restrictions apply to the availability of these data, which were used under license for this study.

## ENDNOTES

^1^
In 2019, the *Centers for Disease Control and Prevention* (CDC) recorded 70,630 drug overdose deaths in the US, 70% of which are due to opioids (Mattson et al., 2021). At their last peak prior to the COVID‐19 pandemic in 2017, opioid overdose deaths were six times higher than in 1999, and have overtaken homicides, suicides, and vehicle accidents as the leading cause of death among Americans below the age of 50 (CDC, 2017). This trend will likely be fueled by the COVID‐19 epidemic. The CDC has not yet released overdose numbers for 2020 and 2021, but Wainwright et al. (2020) report that the presence of fentanyl in random urine samples almost doubled after the national emergency declaration in March 2020, increases in the prevalence of heroin were also noted. Ochalek et al. (2020), analyzing data from a Virginia emergency department, find that the number of nonfatal opioid overdoses increased from 102 in March–June 2019 to 227 in March–June 2020. Initially, opioid abuse was concentrated in low‐income urban areas. Over time, the sociodemographic structure of the epidemic has changed, and opioid addiction has shifted to more affluent suburban and rural areas with primarily white populations (Cicero et al., 2014). Estimates for the total economic burden of prescription opioid abuse alone range up to $78.5 billion per year, including the costs of healthcare and addiction treatment, lost productivity and taxes, as well as law enforcement expenses (Florence et al., 2016).

^2^
See, for example, https://news.un.org/en/story/2019/06/1041341 (accessed June 16, 2020).

^3^
All of these studies exploit data from small‐scale surveys of opioid‐dependent respondents (family history method). The number of respondents varies between 33 and 266. Inference on the intergenerational correlation is based on a comparison of the lifetime prevalence of opioid dependence among the respondents' parents with lifetime prevalence of the general population. In all studies, the former prevalence markedly exceeded the latter, suggesting a positive intergenerational persistence of opioid dependence. A more recent study based on the US National Surveys on Drug Use and Health with 35,000 parent‐child pairs also finds a strong association between self‐reported parental and child use of prescription opioids (Griesler et al., 2019), with effects being stronger for mothers' than fathers' use.

^4^
Despite their obvious public health risks, opioids are aggressively marketed to physicians (Van Zee, 2009). Alpert et al. (2019) show that the pharmaceutical promotion of OxyContin to US physicians explains 65% of the growth in overdose death rates since the 1990s.

^5^
Patients hold mandatory health insurance administered through 9 *Regional Health Insurance Funds* (“Gebietskrankenkassen”), which cover private employees and their dependents, as well as non‐employed individuals. Further 16 social security institutions provide health insurance for specific occupational groups such as farmers, civil servants, and self‐employed persons.

^6^
We argue that the denominator value is, therefore, likely somewhat too high, because only regular users diagnosed with opioid addiction are eligible for substitution therapy.

^7^
Anecdotal evidence we have received from drug counselors suggests that most regular opioid users at some point land in the substitution program, seeking continuous and hassle‐free opioid supply. Occasional usage, so‐called “chipping,” is rare, and is usually an entry point to addiction.

^8^
Upper Austria is one of nine provinces in Austria and comprises about one sixth of the Austrian population and work force. Austria's population is rather homogeneous in terms of demographics (Statistik Austria, 2019), Upper Austria can therefore be considered representative for Austria. The UAHIF covers more than 1 million people, which represent approximately 75% of the Upper Austrian population. The remaining 25% are civil servants, self‐employed, or distinct occupational groups, such as farmers or public teachers (see Endnote 5). We note that this affects the external validity of our findings, because we can only speak to people in regular employment.

^9^
Morphine, for example, is administered both in substitution therapy and in pain treatment. The morphine preparations in ATC category N07BC (*“drugs used in opioid dependence”*) are only approved for substitution therapy, since they come only in dosages that would be far too high for most regular pain patients. The share of prescribed substitution drugs is as follows: buprenorphine (24.9%), morphine (49.7%), methadone (25.4%), naltrexone (0.1%).

^10^
We choose 1984 as the first cohort because we lack socioeconomic information in the birth register prior to that year. A child born in 1984 will be 14 in 1998, a child born in 1990 will be 27 in 2017. In a robustness check, we extend the window to 1980–1990, with a restricted set of covariates, which leads to similar conclusions. Our estimates are also not affected if we control for child year‐of‐birth fixed effects.

^11^
If a child is born during an ongoing marriage, the father is also automatically recorded at the time of birth. If the parents marry at a later point in time, the information on the father is complemented at the time of marriage. Depending on whether the father is linked at birth or at the time of marriage, we have a different set of control variables available. In about 75% of cases, we observe the father link at birth, which results in larger set of covariates.

^12^
Naturally, we can only include family child‐parent pairs in which both are insured with the UAHIF.

^13^
Compliant patients typically remain in substitution therapy for long periods of time, although with several interruptions. Non‐compliant patients who drop‐out of substitution therapy (e.g., due to heroin consumption) typically return at some later point in time. The share of left‐censored observations is 14.6% and 8.8% among mothers and fathers, respectively.

^14^
The medical literature identifies a range of biological and social risk factors associated with opioid addiction. A recent meta analysis identifies previous substance use, the presence of any mental health diagnosis, younger age, and being male as the strongest predictors of opioid abuse (Cragg et al., 2019). Furthermore, specific genetic and epigenetic factors associated with brain reward pathways and impulsivity are linked to addiction vulnerability (Wiss, 2019).

^15^
The reason why we include religious denomination is that we want to control for as many socioeconomic and demographic variables as possible that could potentially affect child opioid use. Religion could play a role in the way norms and values are transmitted across generations, and has been analyzed as a predictor of drug use (e.g., Cheney et al., 2014) or retention in substance abuse treatment (e.g., Shields et al., 2007) before. The religious denomination categories used are a result of the categories available in the data. The data distinguish between Catholics, Protestants, Muslims, Jewish, other denominations, and atheists. These categories reflect the distribution of the most prevalent religious denominations in Austria. Apart from the first three, the only other denomination we could have singled out in our regressions would be Judaism, but only 0.1% of mothers in our cohorts are, in fact, Jewish.

^16^
We note that this does not mean that religion per se affects opioid use—for example, we cannot rule out that being a Muslim is correlated with unobserved confounding factors that drive this correlation.

## Appendix

This Appendix contains additional tables and figures for the paper “*The Intergenerational Persistence of Opioid Dependence: Evidence from Administrative Data*”.

**TABLE A1:** Sample characteristics, cohorts 1984–1990

	Full sample			Illicit opioids^a^			Prescription opioids^b^		
	*n*	Mean	Std. dev.	*n*	Mean	Std. dev.	*n*	Mean	Std. dev.
Substitution status									
Child is substituted between age 14 and 27	81,307	0.011	(0.10)	512	0.070	(0.26)			
Mother is substituted between 1998 and 2017	74,909	0.003	(0.05)	490	0.459	(0.50)			
Father is substituted between 1998 and 2017	60,649	0.005	(0.07)	418	0.720	(0.45)			
Either mother or father is substituted	81,307	0.006	(0.08)	512	1.000	(0.00)			
Average age at onset of substitution therapy									
Child	940	22.230	(3.36)	38	21.406	(3.47)			
Mother	225	41.948	(7.13)	225	41.948	(7.13)			
Father	301	44.727	(6.71)	301	44.727	(6.71)			
Opioid prescription status									
Child takes opioids between age 14 and 27	81,307	0.046	(0.21)				27,053	0.067	(0.25)
Mother takes opioids between 1998 and 2017	81,307	0.204	(0.40)				27,053	0.613	(0.49)
Father takes opioids between 1998 and 2017	81,307	0.177	(0.38)				27,053	0.532	(0.50)
Either mother or father takes opioids	81,307	0.333	(0.47)				27,053	1.000	(0.00)
Average age of first opioid prescription									
Child	4709	23.367	(4.38)				2287	23.324	(4.44)
Mother	16,571	46.365	(7.56)				16,571	46.365	(7.56)
Father	14,401	49.625	(8.24)				14,401	49.625	(8.24)
*N*	81,307			512			27,053		

*Note*: Number of observations for substitution status differs because we assign zeros only when children, mothers, and fathers are insured (children: at age 27; mothers and fathers: at one point between 1998 and 2017). For “either mother or father is substituted,” we assign zeros if either the mother or the father is insured. Age at onset is calculated for all observations that are substituted between 1998 and 2017, which includes also children that were older than 27 at onset of substitution.

^a^ Parents are classified as having had prior opioid abuse.

^b^ Parents were prescribed at least one opioid analgesic between 1998 and 2017.

**TABLE A2:** The intergenerational persistence of opioid dependence: Different levels of clustering

	(1)	(2)	(3)	(4)
(a) Illicit opioids				
Municipality level	0.060*** (0.014)	0.058*** (0.014)	0.057*** (0.014)	0.057*** (0.014)
Child birth characteristics	No	Yes	Yes	Yes
Mother socioeconomics	No	No	Yes	Yes
Environmental conditions	No	No	No	Yes
Family level	0.060*** (0.012)	0.058*** (0.011)	0.057*** (0.011)	0.057*** (0.011)
Child birth characteristics	No	Yes	Yes	Yes
Mother socioeconomics	No	No	Yes	Yes
Environmental conditions	No	No	No	Yes
(b) Prescription opioids				
Municipality level	0.031*** (0.002)	0.031*** (0.002)	0.028*** (0.002)	0.027*** (0.002)
Child birth characteristics	No	Yes	Yes	Yes
Mother socioeconomics	No	No	Yes	Yes
Environmental conditions	No	No	No	Yes
Family level	0.031*** (0.002)	0.031*** (0.002)	0.028*** (0.002)	0.027*** (0.002)
Child birth characteristics	No	Yes	Yes	Yes
Mother socioeconomics	No	No	Yes	Yes
Environmental conditions	No	No	No	Yes

*Note*: This table presents regression results for the determinants of a child using illicit opioid (panel a) and prescription opioids (panel b) before the age of 27 by standard errors clustered at the municipality and family level. The sample is based on the universe of children born between 1984 and 1990. Stars indicate statistical significance: * *p* < 0.10, ** *p* < 0.05, *** *p* < 0.01.

**TABLE A3:** Logistic regressions

	(1)	(2)	(3)	(4)
(a) Illicit opioids				
Intergenerational persistence odds ratio	7.260*** (1.569)	6.170*** (1.335)	5.884*** (1.285)	5.784*** (1.260)
Child birth characteristics	No	Yes	Yes	Yes
Mother socioeconomics	No	No	Yes	Yes
Environmental conditions	No	No	No	Yes
(b) Prescription opioids				
Intergenerational persistence odds ratio	1.932*** (0.055)	1.927*** (0.056)	1.791*** (0.055)	1.761*** (0.053)
Child birth characteristics	No	Yes	Yes	Yes
Mother socioeconomics	No	No	Yes	Yes
Environmental conditions	No	No	No	Yes

*Note*: This table presents logistic regression results for the determinants of a child using illicit opioid (panel a) and prescription opioids (panel b) before the age of 27. The sample is based on the universe of children born between 1984 and 1990. Stars indicate statistical significance: * *p* < 0.10, ** *p* < 0.05, *** *p* < 0.01.
